# Shop till you drop

**DOI:** 10.1007/s12471-020-01478-y

**Published:** 2020-07-31

**Authors:** L. Baris, E. J. van den Bos

**Affiliations:** Department of Cardiology, Albert Schweitzer Hospital, Dordrecht, The Netherlands

A 32-year-old male presented to the emergency department after an episode of vasovagal syncope during a shopping trip with his mother. He had a history of mild aortic regurgitation, but was otherwise healthy. Physical and laboratory examinations did not reveal any abnormalities. The electrocardiogram at presentation is shown in Fig. 1, the electrocardiogram five minutes after presentation is shown in Fig. 2.

**Fig. 1:**
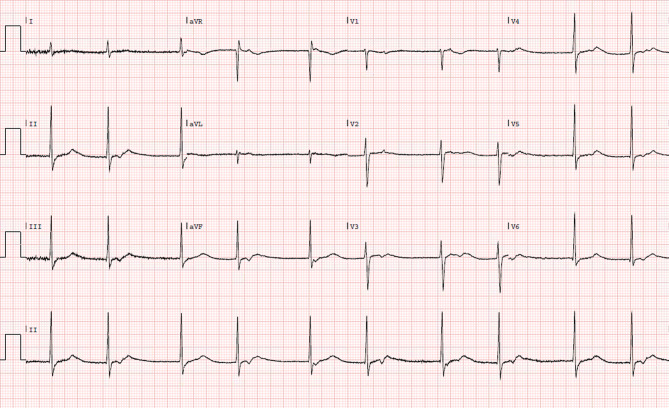
Electrocardiogram at presentation

**Fig. 2:**
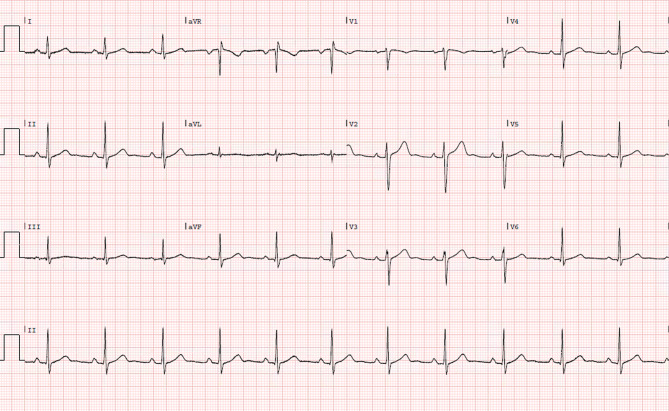
Electrocardiogram 5 min after presentation

What is your diagnosis based on the electrocardiograms?

## Answer

You will find the answer elsewhere in this issue.

